# Progression of Knee Osteoarthritis in Patients Undergoing Medial Opening Wedge High Tibial Osteotomy: A Systematic Review

**DOI:** 10.3390/healthcare14132028

**Published:** 2026-07-07

**Authors:** Michele Mercurio, Federico Piro, Erminia Cofano, Stefano Colace, Filippo Francesco Familiari, Olimpio Galasso, Arianna Carnevale, Umile Giuseppe Longo, Giorgio Gasparini

**Affiliations:** 1 Department of Orthopaedic and Trauma Surgery, “Mater Domini” University Hospital, “Magna Graecia” University, 88100 Catanzaro, Italy; michele.mercurio@unicz.it (M.M.); federico.piro@studenti.unicz.it (F.P.); stefanoleonardo.colace@studenti.unicz.it (S.C.); filippofamiliari@unicz.it (F.F.F.); gasparini@unicz.it (G.G.); 2 Research Center on Musculoskeletal Health, MusculoSkeletal Health@UMG, Magna Graecia University, 88100 Catanzaro, Italy; 3 Department of Medicine, Surgery and Dentistry, University of Salerno, 84081 Baronissi, Italy; ogalasso@unisa.it; 4 Fondazione Policlinico Universitario Campus Bio-Medico, 00128 Rome, Italy; arianna.carnevale@policlinicocampus.it (A.C.); g.longo@policlinicocampus.it (U.G.L.); 5 Research Unit of Orthopaedic and Trauma Surgery, Department of Medicine and Surgery, Università Campus Bio-Medico di Roma, 00128 Rome, Italy

**Keywords:** high tibial osteotomy, revision surgery, survival rate, outcomes, complications, conversion

## Abstract

**Objectives:** Osteoarthritis (OA) of the medial compartment in varus knee is a common degenerative condition. High tibial osteotomy (HTO) has been recognized as a primary joint-sparing procedure. The aim of this systematic review was to investigate functional and radiological outcomes, complications, and the progression of knee OA in patients undergoing medial opening wedge HTO. **Methods:** A total of 18 studies were included. Patients’ demographics, Knee Injury and Osteoarthritis Outcome Score (KOOS), number and types of complications, conversion in arthroplasty surgery rate, survival rate, and radiological evaluation were recorded. **Results:** A total of 2683 patients were evaluated. The frequency-weighted mean follow-up was 168.6 ± 54.6 months. The mean preoperative KOOS score was 46.5 ± 19, while postoperatively the mean score was 66.4 ± 22.9 (*p* < 0.001). The pre- and postoperative mTFA angle was −5.7° ± 2.8°, and 1.4° ± 2.4°, respectively; with a statistically significant improvement (*p* = 0.002). A total of 12.5% of patients had a conversion to a total knee arthroplasty and 1.5% had a conversion to unicompartmental knee arthroplasty, while 1.3% of the patients had a revision surgery unrelated to arthroplasty. The survival rate after 10 years was 86.3%. The nonunion rate was 1.3% and the infection rate was 0.9%. **Conclusions:** Patients who underwent medial opening wedge HTO achieved a significant correction of mTFA associated with an improvement in functional outcomes. An 86% survivorship rate at 10 years was observed, while a 14% conversion rate to knee arthroplasty for OA progression was reported after a mean follow-up of 14 years.

## 1. Introduction

Osteoarthritis (OA) of the medial compartment of the knee is a common degenerative condition that severely affects patients’ quality of life due to chronic pain and functional loss [1]. This pathology is often associated with a varus deformity of the lower limb, which leads to a shift in the weight-bearing distribution to the medial compartment of the knee, thus contributing to the accelerated wear of the articular cartilage [2]. High tibial osteotomy (HTO) has long been recognized as a primary joint-sparing procedure, especially in young and active patients who wish to delay total knee arthroplasty (TKA) [3,4]. The basic biomechanical concept of HTO is the transfer of weight-bearing forces from the affected medial compartment to the less affected lateral compartment by means of the correction of the mechanical axis [5]. Traditionally, the lateral closing wedge high tibial osteotomy was recognized as the gold standard for several decades [6]. However, medial opening wedge high tibial osteotomy has recently gained popularity because of its technical accuracy and the reduced risk of peroneal nerve injury [7]. Recent studies have also analysed the biomechanical consequences of medial opening wedge HTO. Beyond the coronal plane correction, this procedure can influence knee kinematics, joint line alignment, and load distribution. The accuracy of the correction during the surgery is one of the factors that influence postoperative functional outcomes and survivorship. The restoration of the mechanical axis can be considered one of the major factors that ensure unloading of the medial compartment and may contribute to preventing the development of the disease [8]. Another problem is the progression of the patellofemoral arthritis. Despite the successful correction of the varus deformity, the complexity of the biomechanical changes may not completely prevent the degenerative changes of the patellofemoral compartment [9]. These findings suggest that radiographic progression may occur despite satisfactory clinical improvement, highlighting the complexity of the relationship between alignment correction and long-term joint preservation.

In young and active patients, especially those under the age of 45, HTO has shown excellent functional results and long-term clinical stability [10]. However, it has been observed that, despite these early successes, the survival rates of the procedure tend to decline over time [11]. The relationship between alignment correction, radiographic progression, and long-term clinical outcomes remains incompletely understood despite the improvement in pain and function [12]. It has been observed that certain demographic factors, such as a higher body mass index (BMI) and higher ages at the time of surgery, are independent negative predictors of survival rates [13]. An emerging challenge in HTO is the management of residual intra-articular varus [14]. Since HTO is an extra-articular procedure, it may not completely correct intra-articular deformities. In fact, these deformities, as assessed through the joint line convergence angle, may not be completely corrected through HTO [2]. This discrepancy has resulted in OA progression despite a correct limb alignment. Given these complexities, a rigorous analysis of the current evidence is essential if we are to refine patient selection and define the biological and mechanical limits of the procedure [15].

The aim of this systematic review was to investigate functional and radiological outcomes, complications, and the progression of knee osteoarthritis in patients undergoing medial opening wedge high tibial osteotomy.

## 2. Materials and Methods

### 2.1. Search Strategy

A systematic review of the published literature was conducted and reported according to the Preferred Reporting Items for Systematic Reviews and Meta-Analyses statement. The study protocol was registered in PROSPERO (CRD420261387290). The PubMed, MEDLINE, Scopus, and Cochrane Central databases were searched in January 2026. The search terms used to retrieve relevant articles were “tibial osteotomy” AND “varus knee” AND “osteoarthrosis” AND “survival”, AND “outcome”, OR ‘‘results”. Two authors (S.C. and F.P.) independently screened the titles and abstracts to identify articles for inclusion, contacting a third senior author (M.M.) in cases of major discrepancies. The reference lists of each included article, as well as the grey literature available at our institution (including technical reports, theses, institutional documents, and other non-peer-reviewed materials), were screened for potential additional articles.

### 2.2. Inclusion Criteria and Study Selection

The inclusion criteria were applied during title, abstract, and full-text screening according to the PICO: (1) Population—patients who underwent tibial osteotomy for varus knee; (2) Intervention—studies on tibial osteotomy for varus knee reporting >5 surgically treated cases; (3) Comparator—all studies were included, irrespective of the presence or absence of comparator or control groups; (4) Outcome—articles written in English reporting outcomes and/or complications tibial osteotomy for varus knee with a minimum mean follow-up of 12 months. Other reviews, case reports, and articles without outcomes or results, cadaveric or biomechanical studies, technical notes, editorials, letters to the editor, and expert opinions were excluded from the analysis but considered for the Section 4. We excluded studies that do not report explicit clinical, functional, or radiological outcomes, as well as those evaluating alternative surgical procedures.

### 2.3. Data Extraction and Quality Assessment

Two knee surgeons (M.M. and E.C.) examined the included studies and extracted the data. The first author, journal name, year of publication, scientific level, type of surgery, and patient demographics were recorded for each article. Data extracted for quantitative analysis included the Knee Injury and Osteoarthritis Outcome Score (KOOS), the number and types of complications, the conversion in arthroplasty surgery rate, and the survival rate.

Radiologic evaluations of the knee were reported, including assessments of the Kellgren–Lawrence OA classification, the medial Tibiofemoral Angle (mTFA), the medial Lateral Distal Femoral Angle (mLDFA), and the Medial Proximal Tibial Angle (MPTA).

A methodological quality assessment was conducted independently by two authors (M.M. and E.C.); cohort studies were assessed using the Modified Newcastle–Ottawa Quality Assessment Scale [16]. Randomized controlled trials were assessed with version 2 of the risk of bias tool [17], as recommended by the Cochrane Collaboration. Details of the quality assessment are shown in Table 1.

### 2.4. Statistical Analysis

The quantitative data were organized for statistical analysis; all data were collected, measured, and reported with 1-decimal accuracy. Weighted means and standard deviations were calculated for data concerning demographic characteristics and outcomes. When standard deviations were not directly provided, they were calculated with the equation [max range − min range/4] to allow for statistical aggregation. The normality of data distribution was evaluated using the Shapiro–Wilk test. Continuous variables representing paired pre- and postoperative data were analysed using the non-parametric Wilcoxon signed-rank test to minimize the risk of type-II errors. Proportions were analysed using 2 × 2 contingency tables [18]. All tests were performed with SPSS Statistics software (version 25.0; IBM Corp., Armonk, NY, USA) and GraphPad Prism (version 7.0; GraphPad Software Inc., San Diego, CA, USA). Confidence intervals were set at 95%, and a *p* value less than 0.05 was considered significant.

**Table 1:** Quality assessment of included studies according to the Modified Newcastle–Ottawa Quality Assessment Scale.

Study Author (Year)	Criteria								Tot.	Quality
	1	2	3	4	5	6	7	8		
Ahrend M.C (2025) [19]	2	0	2	1	1	1	1	1	8	High
Akizuki S. (2008) [20]	2	0	2	1	1	1	1	1	9	High
Benzakour T. (2010) [21]	2	0	2	1	1	1	1	1	9	High
Choi H. R. (2001) [22]	1	0	2	1	1	1	1	1	8	High
Dal Fabbro (2025) [7]	1	0	2	1	1	1	1	1	8	High
E. Hantes (2017) [10]	1	0	2	1	1	1	1	1	8	High
Flecher X. (2006) [23]	2	0	2	1	1	1	1	1	9	High
Gkekas N. K. (2024) [15]	1	0	2	1	1	1	1	1	8	High
Michaela G. (2008) [24]	2	0	2	1	1	1	1	1	9	High
Howells N. R. (2014) [25]	2	0	2	1	1	1	1	1	9	High
Hui C. (2010) [26]	2	0	2	1	1	1	1	1	9	High
Koshino (2004) [14]	1	0	2	1	1	1	1	1	8	High
Leutloff D. (2001) [27]	1	0	2	1	1	1	1	1	8	High
Mabrouk A. (2023) [28]	2	0	2	1	1	1	1	1	9	High
Pfahler (2003) [29]	1	0	2	1	1	1	1	1	8	High
Marti RK. (2001) [3]	1	0	2	1	1	1	1	1	8	High
Valenti J. R. (1990) [30]	1	0	2	1	1	1	1	1	8	High
van Wulfften Palthe (2018) [11]	2	0	2	1	1	1	1	1	9	High

Based on the total score, quality was classified as “low” (0–3), “moderate” (4–6) or “high” (7–9). Criterion number (in bold): 1, representativeness of the exposed cohort; 2, selection of the nonexposed cohort; 3, ascertainment of exposure; 4, demonstration that outcome of interest was not present at start of study; 5, comparability of cohorts on the basis of the design or analysis; 6, assessment of outcome; 7, determining whether follow-up was long enough for outcomes to occur; 8, adequacy of follow-up of cohorts. Each study was awarded a maximum of one or two points for each numbered item within categories, based on the Modified Newcastle–Ottawa Quality Assessment Scale rules.

## 3. Results

**Figure 1:**
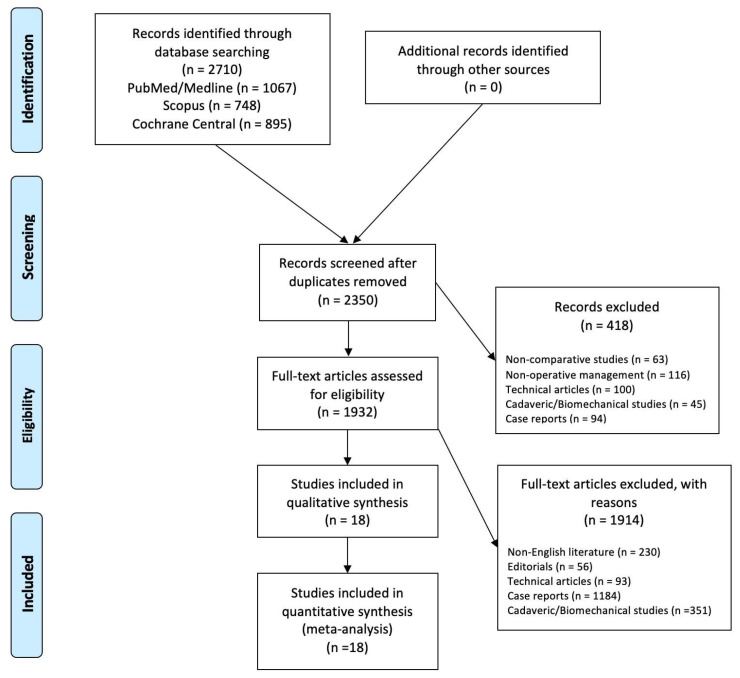
Preferred Reporting Items for Systematic Review and Meta-Analysis (PRISMA) flowchart for the searching and identification of included studies. Source: Moher et al. For more information, visit www.prisma-statement.org.

A total of 2710 relevant articles were identified through the initial search, 2350 abstracts were screened, and 1932 full-text articles were assessed for eligibility based on our inclusion criteria, resulting in 18 studies that were eligible for the systematic review (Figure 1).

A total of 2683 patients were identified; overall, male patients accounted for 1628 (60.7%) of the cases. The frequency-weighted mean age at the time of the operation was 49.9 ± 11.9 years, and the frequency-weighted mean follow-up was 168.6 ± 54.6 months. The weighted mean BMI was 27.7 ± 5.4 kg/m^2^. The characteristics of the included studies are reported in Table 2.

### 3.1. Functional Outcomes

The pre- and postoperative KOOS scores were measured in three studies with 703 patients, with mean weighted values of 46.5 ± 19 and 66.4 ± 22.9, respectively; a statistically significant improvement was found (*p* < 0.001).

### 3.2. Radiological Evaluation

The preoperative and postoperative radiological evaluations are shown in Table 3.

According to the Kellgren–Lawrence classification, a total of 6 patients had Grade 0 (0.7%), 78 patients (8.9%) had Grade 1, 324 patients (36.8%) had Grade 2, 307 patients (34.9%) had Grade 3, and 165 patients (18.7%) had Grade 4.

The pre- and postoperative mTFA angles were measured in four studies with 798 patients, with mean weighted values of −5.7° ± 2.8° and 1.4° ± 2.4°, respectively; a statistically significant improvement was found (*p* = 0.002).

The pre- and postoperative mLDFA angles were measured in three studies with 816 patients, with mean weighted values of 88.8° ± 1.9° and 88.7° ± 2°, respectively (*p* = 0.3).

The pre- and postoperative MPTA angles were measured in four studies with 848 patients, with mean weighted values of 90.7° ± 7.5° and 90.8° ± 3.3°, respectively (*p* = 0.722).

### 3.3. Revision, Conversion to Arthroplasty, and Survivorship

The characteristics of surgery are shown in Table 4. A total of 335 patients (12.5%) had a revision surgery with a conversion to a total knee arthroplasty (TKA), while a total of 41 (1.5%) had a conversion to unicompartmental knee arthroplasty (UKA).

A total of nine studies reported the survival rate after 5 years, with a mean rate of 94.3% (1843 patients).

A total of 10 studies reported the survival rate after 10 years, with a mean rate of 86.3% (1828 patients).

### 3.4. Complications

Nonunion rate was 1.3%, while peroneal nerve palsy rate was 1.3%. The infection rate was 0.9% (25 patients). Superficial wound infection was reported in 26 patients, comprising 1%. Finally, deep venous thrombosis was reported in 22 patients (0.8%). The results are shown in Table 5.

## 4. Discussion

Medial opening wedge high tibial osteotomy achieved a statistically significant correction of the mTFA with improvements in the functional outcomes. The procedure demonstrated a 10-year survival rate of 86%, with a 14% conversion rate to total or unicompartmental knee arthroplasty for OA progression after a mean follow-up of 14 years.

HTO is still considered a primary indication for the treatment of medial compartment knee OA in young and active patients, by acting as a joint-preserving strategy that maintains native joint kinematics [15]. Unlike arthroplasty, HTO preserves native ligaments and bone stock, maintaining proprioception and allowing higher postoperative activity levels with a more physiological knee perception. Several studies have also shown that opening wedge HTO remains an effective option even in patients with advanced OA, challenging the traditional consideration that this procedure should be used for early-stage OA [7,15,19,31].

Correction of the mTFA remains the main biomechanical determinant of HTO success, and its loss over time represents one of the most relevant mechanisms underlying long-term failure. Our results move from −5.7° to 1.4°, which is consistent with the recommendations of Koshino et al. [14], who emphasized that achieving and maintaining a valgus alignment is the most significant factor in preventing the recurrence of varus deformity and OA progression. Also, maintaining stable joint kinematics is important to avoiding complications in further surgical interventions [7,32]. The survival of HTO appears to depend not only on the degree of correction achieved but also on the long-term maintenance of both coronal and sagittal alignment. In fact, Dal Fabbro et al. [7] discussed the importance of preserving the posterior tibial slope (PTS) because a change in the slope may adversely affect joint biomechanics and accelerate the progression of OA [33]. Similarly, Choi et al. [22] reported a progressive relapse to varus alignment after 15 years; the average Tibiofemoral Angle varies from 187° to 170°, suggesting that the loss of correction may contribute to long-term failure. This mechanism may partially explain the 14% osteotomy failure rate observed in our review, consistent with the 14% revision rate reported by Flecher et al. [23].

The radiological correction obtained is associated with the enhanced functional outcomes, which are consistent findings across the literature. Mabrouk et al. [28] and Ahrend et al. [19] reported high patient satisfaction and significant improvements in quality-of-life scores, maintained over 10 years postoperatively. In our study, KOOS significantly improved from 46.5 ± 19 preoperatively to 66.4 ± 22.9 postoperatively (*p* < 0.001), with a mean increase of 19.9 ± 4 points, consistently exceeding the Minimal Clinically Important Difference (MCID) threshold of 10 points [34]. These results are particularly relevant considering the relatively young age of our cohort (49.9 years), as they represent a population that prioritizes the ability to remain active [35]. The partial dissociation between radiographic results and patient-reported outcomes was found. Some studies have found patients who have shown a sustained improvement clinically while having radiographic progression. It suggests that the alleviation of symptoms achieved by HTO may be driven by biomechanical unloading of the medial compartment rather than complete arrest of the degenerative process.

In this context, patient selection remains a crucial factor that influences the success of HTO. Several studies demonstrated that younger age, lower BMI, and limited cartilage degeneration are associated with higher outcomes and survivorship. Higher BMI may increase mechanical load on the osteotomy site, making accurate correction more challenging. These findings underline the importance of careful preoperative assessment in optimizing patient selection, which remains one of the most important determinants of long-term success [36,37].

In our study, the survival rate at 10 years was 86.3%. Michaela et al. [30] reported a lower survival rate of 79.9% at 10 years, which dropped significantly to 54.1% after 18 years. In contrast, Flecher et al. [23] demonstrated high longevity with 85% survival at 20 years, consistent with the data reported by Akizuki et al. [20], who also confirmed stable outcomes in their 10-to-20-year follow-up period. These observations suggest that the precision of the correction may be more critical than the specific surgical approach used. The variability in survivorship reported across the included studies reflects the differences in patient selection, correction targets, and OA severity at the time of surgery. Studies including younger patients with lower BMI and minor cartilage degeneration generally reported higher long-term survival rates.

Despite these favourable outcomes, HTO should still be considered a time-buying procedure. In our cohort, 14% of patients required a conversion to knee arthroplasty, which demonstrates the necessity of using this approach as a bridge. This transition to TKA remains a critical aspect. The meta-analysis of Loke et al. [38] (which reviewed more than 550,000 patients) found that, although HTO and subsequent TKA result in similar survival and functional results to those of primary TKA, it is associated with significantly higher complication rates. This emphasizes the importance of the surgical technique during the initial HTO, particularly regarding hardware placement and preservation of posterior tibial slope, in order to reduce technical difficulties during eventual conversion to TKA [7].

Finally, our findings align with the evidence provided by Ge et al. [39], who confirmed that HTO results in approximately 14 years of survivorship for young patients with active lifestyles. However, this finding should be interpreted within the context of the relatively young age of the treated population. Delaying arthroplasty by more than a decade may represent a clinically meaningful achievement, potentially reduce the lifetime risk of revision arthroplasty while enabling patients to maintain higher activity levels during the most active years of their lives.

Although the survivorship, HTO remains associated with relevant complications. In our study, nonunion and peroneal nerve palsy were both observed in 1.3% of cases, while the overall infection rate was 0.9%. Revision surgery unrelated to arthroplasty conversion was required in 1.3% of patients. These findings are similar to the complication rate reported in the previous reviews. In the systematic review by Miltenberg et al. [40], which analysed 7836 patients, an overall postoperative complication rate of 6.9% was reported; with superficial wound infection representing the most common one (2.2%), followed by nonunion (1.9%). The authors also described an overall reoperation rate of 15.5%.

Bone healing complications remain the most clinically relevant adverse event, as the instability at the osteotomy site may contribute to delayed union, loss of correction, or fixation failure. Neurological and thromboembolic complications, although relatively uncommon, may also impact postoperative recovery and patient morbidity. In our cases, the complication rates are in line with the literature, suggesting that meticulous surgical technique, stable fixation, and postoperative management are essential for minimization.

This study has several limitations. First, only English language studies were considered, potentially contributing to publication bias; moreover, although four recommended databases were used for the search, we cannot exclude the possibility that additional articles could have been found by searching other databases. Second, heterogeneity of the technique across the included cohorts with different fixation devices makes it difficult to generalize specific complication rates. Finally, the lack of detailed data on modifiable biological factors, such as smoking status, limits the possibility of a more precise risk stratification.

These findings mean that HTO is not seen just as a temporary procedure, but rather a long-term strategy that effectively redefines patients’ surgical timelines, offering a reliable decade of high-level knee function.

## 5. Conclusions

This systematic review showed that patients who underwent medial opening wedge high tibial osteotomy achieved a significant correction of mTFA associated with an improvement in the functional outcomes. An 86% survivorship rate at 10 years was observed, while a 14% conversion rate to knee arthroplasty for OA progression was reported after a mean follow-up of 14 years. Low incidence rates for nonunion (1.3%) and infection (0.9%) were found. The results of the current study may be of interest to patients, clinicians, and health professionals involved in the management of under-50 and active patients with medial compartment OA in a varus knee.

## Figures and Tables

**Table 2:** Characteristics of included studies.

Authors	Journal	Year of Publication	LOE	Patients Demographics										
				Number of Patients	Sex		Age (Years)			BMI		Follow-Up (Months)		
					M	F	Mean	Range	SD	Mean	SD	Mean	Range	SD
Ahrend et al. [19]	*Knee surgery, sports traumatology, arthroscopy*	2025	IV	95	67	28	47	19.2–61.7	7.6	NA	NA	154.8	144–181.2	9.6
Akizuki et al. [20]	*The journal of Bone & Joint Surgery*	2008	IV	132	20	112	62.9	45–76	7.75	NA	NA	196.8	192–240	12
Benzakour et al. [21]	*International Orthopaedics*	2010	IV	192	58	134	55	40–72	8	NA	NA	180	60- 324	66
Choi et al. [22]	*Journal of Orthopaedic Science*	2001	IV	26	8	18	59	48–70	5.5	NA	NA	183.6	NA	42
Dal Fabbro et al. [7]	*International Orthopedics*	2025	V	70	62	8	43.4		10.6	25.3	3.6	133.2		43.2
Flecher et al. [23]	*Clinical orthopaedics and related research*	2006	IV	313	194	119	42	15–76	15.25	24.9	5.8	216	244–336	23
Gkekas et al. [15]	*Knee surgery, sports traumatology, arthroscopy*	2024	IV	32	27	5	47.1	30–57	9.17	NA	NA	163.2	84–240	39
Michaela et al. [24]	*The Archives of Orthopaedic and Trauma Surgery*	2008	IV	111	58	53	54.5	19–74	13.75	NA	NA	148.8	12–300	72
Hantes et al. [10]	*Knee Surgery, Sports Traumatology, Arthroscopy*	2017	IV	20	18	2	35.4	28–44	4	NA	NA	147.6	120–180	15
Howells et al. [25]	*The journal of Bone & Joint Surgery*	2014	III	95	69	26	50	26–66	10	28	4.5	120	NA	NA
Hui et al. [26]	*The American Journal of Sports Medicine*	2010	IV	413	326	87	50	24–70	11,4	NA	NA	180	60–324	66
Koshino et al. [14]	*Science Direct*	2004	IV	53	11	42	59.6	46–73	6.7	25.3	3.6	228	180–336	36
Leutloff et al. [27]	*International Orthopaedics*	2001	III	53	29	24	38	17–73	14	NA	NA	126	69.6–199.2	32.4
Mabrouk et al. [28]	*Knee surgery, sports traumatology, arthroscopy*	2023	IV	651	462	189	46.7	37.7–55.7	3	29.6	5.2	158.1	NA	45.4
Pfahler et al. [29]	*Acta Chirurgia Belgica*	2003	IV	91	50	41	54	20–67	11.75	NA	NA	122.4	72–168	24
Marti et al. [3]	*The journal of Bone & Joint Surgery*	2001	IV	34	12	22	43	17–66	13.6	NA	NA	132	60–252	48
Valenti et al. [30]	*International Orthopaedics*	1990	IV	79	34	45	56	17–74	14.25	NA	NA	132	96–196	25
Van Wulfften Palthe et al. [11]	*European Journal of Orthopaedic Surgery & Traumatology*	2018	III	223	123	100	54	24–80	14	27.2	3.9	144	NA	48

LOE, level of evidence; SD, standard deviation; BMI, body mass index; NA, not available.

**Table 3:** Pre- and postoperative radiographical evaluations.

	Kellgren–Lawrence					mTFA				mLDFA				MPTA			
	Grade 0	Grade 1	Grade 2	Grade 3	Grade 4	Pre-op		Post-op		Pre-op		Post-op		Pre-op		Post-op	
						Mean (°)	SD	Mean (°)	SD	Mean (°)	SD	Mean (°)	SD	Mean (°)	SD	Mean (°)	SD
Ahrend et al. [19]	0	1	62	21	11	−5.1	2.3	2	2.3	89.1	2	89.2	1.8	86.1	2.7	85.7	2.5
Dal Fabbro et al. [7]	0	6	19	31	15	NA	NA	NA	NA	89.7	3	90	1.8	82.2	4.4	90.3	3.7
Gkekas et al. [15]	NA	NA	NA	21	14	−7.8	2.4	2.8	1.9	NA	NA	NA	NA	83.1	2	90.1	2
Hantes et al. [10]	NA	NA	NA	NA	NA	−5.8	2.4	2.5	1.9	NA	NA	NA	NA	NA	NA	NA	NA
Koshino et al. [14]	0	0	40	24	4	NA	NA	NA	NA	NA	NA	NA	NA	NA	NA	NA	NA
Mabrouk et al. [28]	6	71	203	210	121	−5	2.9	1.3	2.5	88.7	1.8	88.5	2	92.7	2.2	91.6	2.7
Total	6	78	324	307	165	−5.7	2.8	1.4	2.4	88.8	1.9	88.7	2	90.7	7.5	90.8	3.3

SD, standard deviation; NA, not applicable.

**Table 4:** Characteristics of surgery of the included studies.

Authors	Conversion to TKA		Conversion to UKA		Survival at 5 Years	Survival at 10 Years
	N	%	N	%	%	%
Ahrend et al. [19]	29	30.5	2	2.1	88.2	76
Akizuki et al. [20]	11	9.3	NA	NA	99	98
Benzakour et al. [21]	23	10.3	NA	NA	NA	NA
Choi et al. [22]	4	13.3	NA	NA	NA	NA
Dal Fabbro et al. [7]	6	4.2	NA	NA	NA	NA
Flecher et al. [23]	20	6.5	13	4.2	95	93
Gkekas et al. [15]	3	8.6	NA	NA	NA	92
Michaela et al. [24]	53	39.6	NA	NA	94	80
Hantes et al. [10]	1	5	NA	NA	NA	NA
Howells et al. [25]	15	15.7	5	5.3	87	79
Hui et al. [26]	NA	NA	NA	NA	95	79
Koshino et al. [14]	NA	NA	NA	NA	97.8	96
Leutloff et al. [27]	5	8.8	NA	NA	NA	NA
Mabrouk et al. [28]	38	5.8	21	3.2	97.2	92
Pfahler et al. [29]	24	38.7	NA	NA	74	NA
Marti et al. [3]	NA	NA	NA	NA	NA	NA
Valenti et al. [30]	5	5	NA	NA	NA	NA
Van Wulfften Palthe et al. [11]	98	44	NA	NA	NA	75
Total	335	12.5	41	1.5	94.3	86.3

TKA, total knee arthroplasty; UKA, unicompartmental arthroplasty; N, number; NA, not applicable.

**Table 5:** Complications.

Authors	Infection		Nonunion		Peroneal Nerve Palsy		Superficial Wound Infection		Deep Venous Thrombosis	
	N	%	N	%	N	%	N	%	N	%
Mabrouk et al. [28]	23	3.5	7	1	1	0.15	11	1.7	1	0.15
Marti et al. [3]	1	3	NA	NA	3	9	1	3	NA	NA
Dal Fabbro et al. [7]	NA	NA	NA	NA	NA	NA	3	4.3	NA	NA
Gkekas et al. [15]	NA	NA	NA	NA	NA	NA	NA	NA	NA	NA
Koshino et al. [14]	NA	NA	NA	NA	3	5.7	NA	NA	NA	NA
Ahrend et al. [19]	NA	NA	NA	NA	NA	NA	NA	NA	NA	NA
Akizuki et al. [20]	NA	NA	2	1.6	5	4.2	1	0.8	1	0.8
Choi et al. [22]	NA	NA	NA	NA	1	3.8	NA	NA	NA	NA
Flecher et al. [23]	1	0.3	1	0.3	1	0.3	NA	NA	2	0.6
Michaela et al. [24]	NA	NA	19	17.1	7	6.3	2	1.8	10	9
Howells et al. [25]	NA	NA	3	3.15	1	1	2	2.1	NA	NA
Hui et al. [26]	NA	NA	1	0.24	1	0.24	NA	NA	NA	NA
Leutloff et al. [27]	NA	NA	2	3.8	4	7.5	2	3.8	4	7.5
Pfahler et al. [29]	NA	NA	NA	NA	4	4.4	NA	NA	1	1.1
Valenti et al. [30]	NA	NA	NA	NA	NA	NA	NA	NA	NA	NA
Van Wulfften Palthe et al. [11]	NA	NA	NA	NA	NA	NA	NA	NA	NA	NA
Benzakour et al. [21]	NA	NA	NA	NA	3	1.5	4	2	3	1.5
Hantes et al. [10]	NA	NA	NA	NA	NA	NA	NA	NA	NA	NA
Total	25	0.9	35	1.3	31	1.3	26	1	22	0.8

TKA, total knee arthroplasty; UKA, unicompartmental arthroplasty; N, number; NA, not applicable.

## Data Availability

No new data were created or analysed in this study.
